# Management of a contained ruptured infrarenal abdominal aortic pseudoaneurysm caused by inferior vena cava struts injury: A case report and literature review

**DOI:** 10.1016/j.jvscit.2021.03.002

**Published:** 2021-05-21

**Authors:** Emilie Robinson, Lia Jordano, Aleem K. Mirza, Laura Eisenmenger, Nedaa Skeik, Jesse Manunga

**Affiliations:** a Section of vascular & Endovascular Surgery, Minneapolis Heart Institute at Abbott Northwestern Hospital, Minneapolis, Minn; b Division of Neuroradiology, Department of Radiology, University of Wisconsin at Madison, Madison, Wisc

**Keywords:** Aortic pseudoaneurysm, Endovascular repair, IVC filter, Penetrating aortic ulcer

## Abstract

Aortic pseudoaneurysms are rare entities caused by infection, trauma, atherosclerotic plaque rupture, or aortic instrumentation. Their natural course remains unknown; however, repair is invariably recommended. We present a case of a 71-year-old man with a history of recurrent deep venous thrombosis and pulmonary embolisms who underwent an inferior vena cava filter placement 8 years prior and was found to have a 3.6-cm contained ruptured infrarenal aortic pseudoaneurysm on imaging performed for abdominal pain. His pseudoaneurysm was excluded using a Gore Excluder Endoprosthesis. We further reviewed literature on the subject to highlight the various surgical approaches to this lethal condition.

In patients who have failed or those with contraindications to anticoagulation, inferior vena cava filters (IVCF) represent a useful prophylactic modality for the prevention of pulmonary embolus (PE), although it is not without potential complications, including perforation.^1^^,^^2^ In fact, some degree of caval wall penetration is inherent to the filter design and is intended to prevent migration. However, penetration of more than 3 mm is considered pathologic and reported to occur in 9% to 24% of cases.^2^ Although the majority of cases are asymptomatic or clinically insignificant, subsequent aortic erosion and pseudoaneurysm represents a rare but serious complication and often requires operative management.^2^^,^^3^ According to manufacturer and user facility device experience database, vena cava perforation represents only 20% of all IVCF-related complications. Injuries to adjacent structures such as lumbar arteries, aorta, iliac arteries, duodenum, renal pelvis, and ureter have been reported. However, these complications are uncommon.^1^^,^^3^, ^4^, ^5^, ^6^, ^7^, ^8^, ^9^, ^10^, ^11^ Management for most these complications traditionally involved open surgical reconstruction and removal of the filter. However, in cases involving the aorta and where infection can be ruled out, endovascular intervention should be considered given its advantages over open aortic reconstruction.^12^^,^^13^

We present a rare complication of an aortic pseudoaneurysm (APA) secondary to erosion of an Eclipse IVCF (Bard, Tempe, Ariz) that was successfully excluded with a Gore Excluder Endoprosthesis. To provide a more complete picture on the management of this rare complication, we performed a PubMed advanced search with the following search terms designed to be inclusive of IVCF aortic complications: ((IVC filter) OR (inferior vena cava filter)) AND ((aorta) OR (aortic) OR (penetration) OR (perforation) OR (rupture) OR (aneurysm) OR (pseudoaneurysm)). The search generated 584 publications, 40 of which had a true IVCF-related aortic injury and 6 publications, all of which were case reports, had the specific complication of APA resulting from filter penetration of the aortic wall (Fig 1 and Table). The patient consented to the publication of the case details and images.

**Fig 1:**
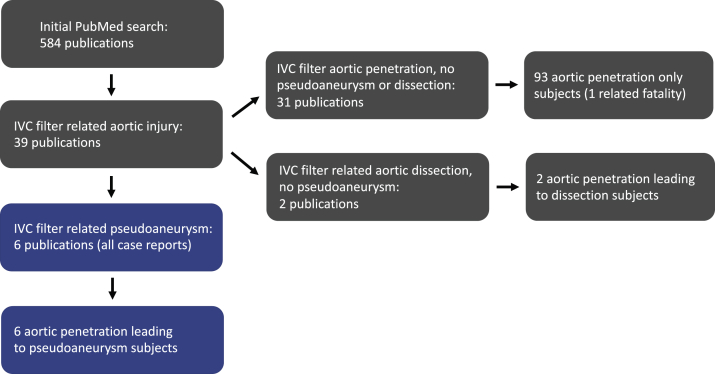
Flow chart of articles from advanced PubMed search of the following terms: ((IVC filter) OR (inferior vena cava filter)) AND ((aorta) OR (aortic) OR (penetration) OR (perforation) OR (rupture) OR (aneurysm) OR (pseudoaneurysm)). The most common IVC filter related aortic injury was asymptomatic aortic wall penetration; however, this search yielded six cases of filter prong penetration associated pseudoaneurysm formation. *IVC*, inferior vena cava.

**Table:** Literature review

Publication year	Reference	Age/sex	Filter	Time lapse	Symptomatic	Associated injury	Infected	Repair	Stent	Filter removed
2003	Campbell and Calcagno^6^	29 M	Bird's Nest	1 years	Yes	No	Yes	Staged axillary-femoral bypass, aortoiliac ligation	–	No
2005	Putterman et al^8^	50 F	Simon Nitinol	7 years	Yes	No	No	Endovascular stent graft	Wallgraft + Wallstent Boston Scientific	No
2006	Medina et al^3^	64 F	Bird's Nest	10 years	Yes	No	No	EVAR	Cook Zenith	No
2010	Becher et al^7^	42 F	Celect R- IVCF	10 months	Yes	Duodenum	Yes	Excision, vein interposition graft	–	Yes
2012	Assifi et al^10^	34 M	Celect	9 months	Yes	Duodenum Iliopsoas	Yes	Excision, homograft interposition	–	Yes
2019	McEnulty et al^11^	24 M	Unknown	3 years	Yes	Duodenum	Yes	EVAR → open repair	Unknown	No

*EVAR,* Endovascular aneurysm repair; *IVCF,* inferior vena cava filter.

Case reports of all patients with aortic pseudoaneurysm related to IVC filter struts. Interestingly, four of the six patients were infected. Note that all of these filters have hooks at the endo of their prongs. Although speculative, it is conceivable that this feature makes them more likely to erode into the wall of the vena cava and surrounding structures when left in place for a long time.

## Case report

A 71-year-old man with cardiovascular risk factors significant for morbid obesity, diabetes mellitus type 2, hypertension, hyperlipidemia, Crohn's disease, deep vein thrombosis (DVT), multiple, pulmonary embolisms and an IVCF placement 8 years prior was referred to vascular surgery for management of 3.3 × 3.6 cm contained ruptured APA. The patient underwent a C.R. Bard Eclipse placement in 2011 after being admitted with a recurrent DVT, pulmonary embolisms, and multiple lower extremity hematomas while on coumadin. His medication was switched to apixaban during the index hospitalization, and he has not suffered another PE since.

**Fig 2:**
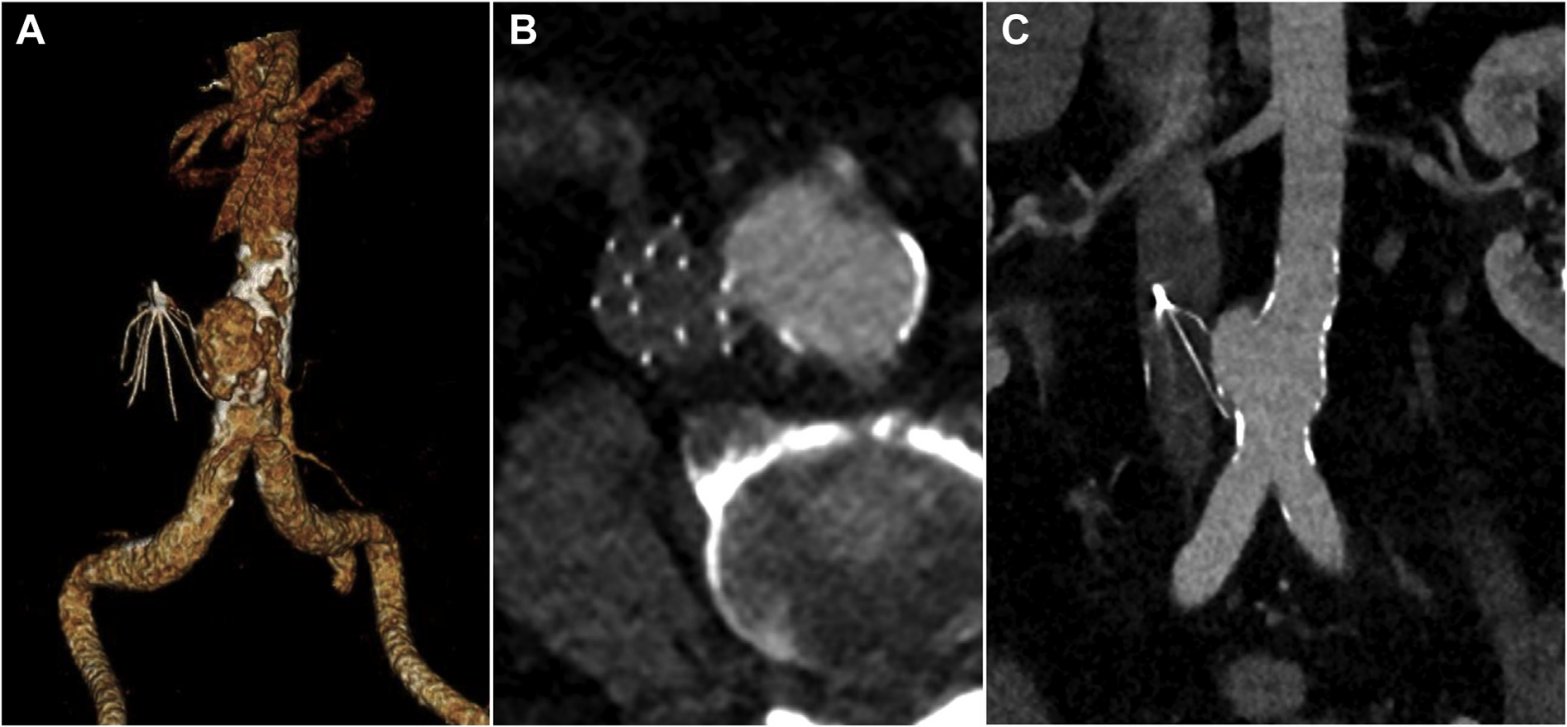
Preoperative computed tomography angiography (CTA). **(A)** CTA reconstruction, **(B)** Axial view of the CTA and **(C)** coronal view of the CTA showing the aortic pseudoaneurysm (APA) and a C.R. Bard Eclipse inferior vena cava filter (IVCF). The struts or prongs of the IVCF extend beyond the margins of the IVC, penetrating the wall of the APA. **(B, C)** There is a paucity of calcifications involving the pseudoaneurysm, which can be a sign of the aortic wall injury with focal expansion, or pseudoaneurysm formation, leading to separation of the atherosclerotic calcifications. Separation or a focal defect in atherosclerotic calcifications has also been associated with cases of impending aortic rupture. The patient had an extensive infectious workup and had a normal C-reactive protein, negative blood culture, no leukocytosis or fevers and CT findings were not concerning for infection.

A computed tomography angiography (CTA) in late 2018 revealed a protrusion of the struts of the IVCF with encroachment on the aortic wall and surrounding inflammatory changes. Because he was asymptomatic and a review of prior imaging revealed no change in the appearance over the preceding years, he was monitored. A CTA performed 2 years later for abdominal pain revealed a focal penetration of the aorta with pseudoaneurysm formation measuring 3.3 × 3.6 cm in diameter at the level of the inferior mesenteric artery (IMA) takeoff (Fig 2). The infectious workup was negative. The patient refused IVCF removal, but consented to endovascular repair of the APA.

The procedure was performed in a hybrid room under general anesthesia. Systemic anticoagulation with bivalirudin was initiated after gaining percutaneous bilateral common femoral artery access. Aortogram confirmed the presence of the APA (Fig 2, *A*). The IMA was selectively cannulated and embolized using Nester coils (Cook Medical, Bloomington, Ind) while preserving collateral flow via a branch to the internal iliac artery and arc of Riolan.

**Fig 3:**
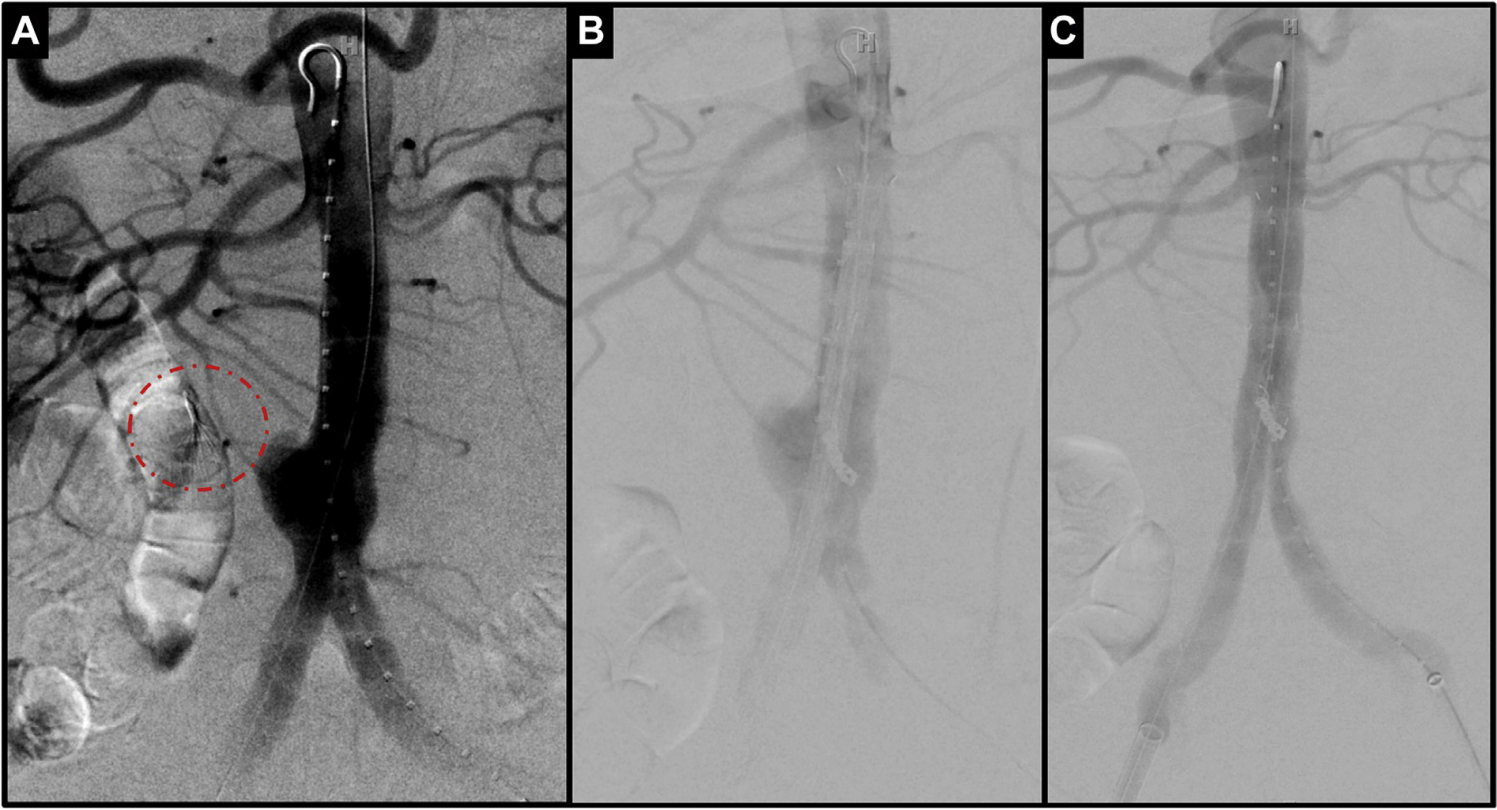
Intraoperative images including **(A)** before and **(B)** after inferior mesenteric artery (IMA) embolization and stent graft placement and **(C)** completion angiography showing excellent exclusion of the aneurysm with no endoleak.

**Fig 4:**
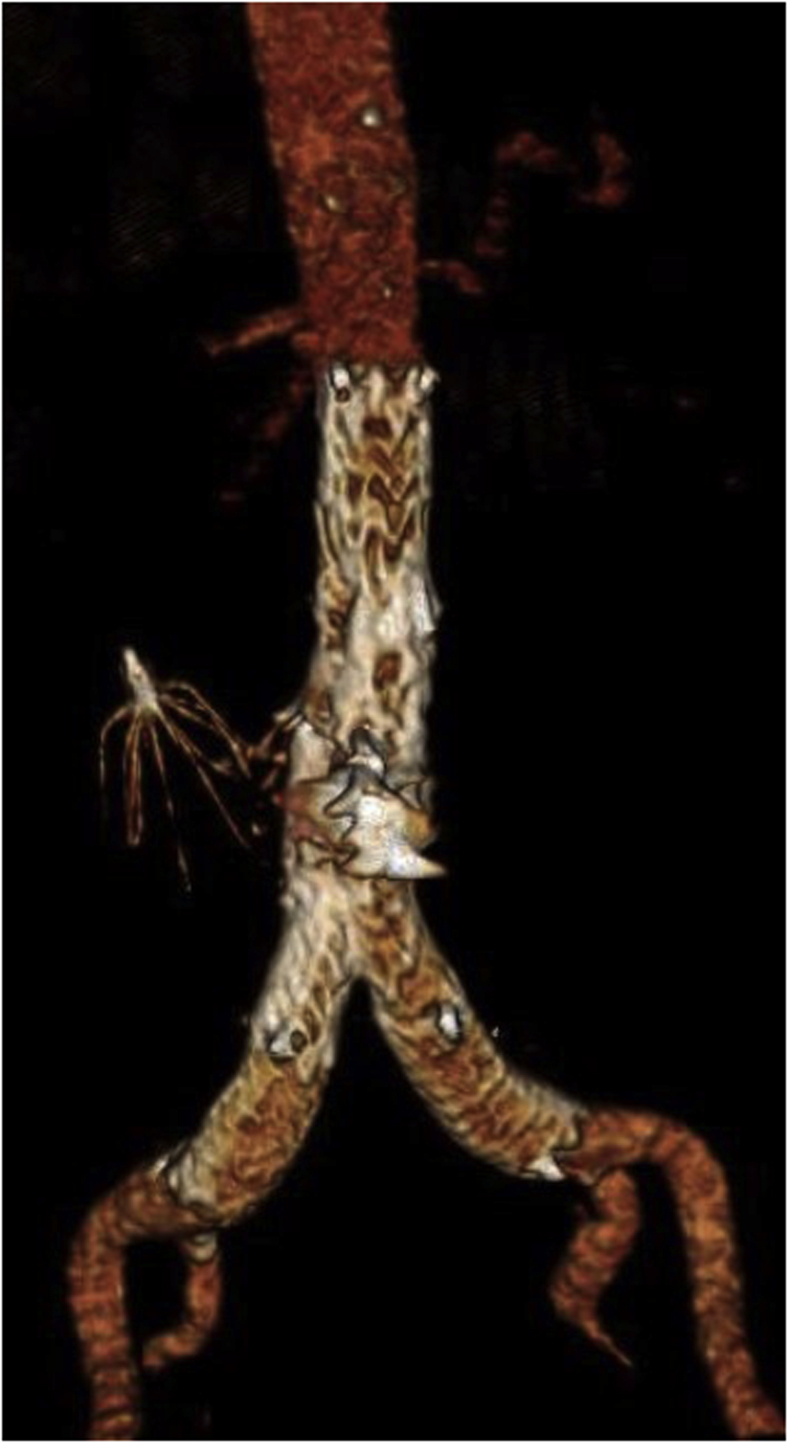
Postoperative computed tomography angiography (CTA) reconstruction of the aorta showing exclusion of the pseudoaneurysm with a stent graft.

To avoid contralateral gate compression and difficulty cannulating in a nonaneurysmal aorta, we elected to precannulate the gate using a technique we have recently reported (Fig 3, *B*).^14^ The remainder of the procedure was carried out in the standard fashion using a 23 × 14.5 × 120-mm Excluder Endoprosthesis and 18-mm limbs (W. L. Gore & Associates, Flagstaff, Ariz). Completion angiography demonstrated excellent exclusion of the pseudoaneurysm with no endoleak (Fig 3, *C*). The patient's pain resolved and he was discharged home on postoperative day 1. The 1-month follow-up CTA demonstrated the endograft in good position, no flow within the pseudoaneurysm, and no endoleak (Fig 4).

## Discussion

IVCF removal is recommended in patients with filter-related complications after weighing the risks and benefits of such an intervention.^15^ For this reason, the majority of filters used in clinical practice are removable and can readily be retrieved by endovascular means. However, of the six cases reviewed in this article, not a single patient underwent an endovascular filter retrieval in conjunction with pseudoaneurysm repair. Two filters were removed after open aortic reconstruction. In both cases, struts were also penetrating the duodenum and patients were septic.^7^^,^^10^ The third patient with a pseudoaneurysm and duodenal perforation underwent an endovascular aneurysm repair to temporize a ruptured pseudoaneurysm before being transferred to another center for open repair. It is unknown whether the filter was removed during the aortic reconstruction. However, it is safe to assume it was because the duodenum was involved and the patient was septic.^11^ One patient with no infection underwent open repair consisting of staged axillary-femoral-femoral bypass followed by aortic ligation without filter removal.^6^ Endovascular therapy was offered to two patients, using a bifurcated device in one and a Wallgraft in another.^3^^,^^8^ We opted for an endovascular repair with a bifurcated device after IMA embolization because the pseudoaneurysm was only 8 mm above the aortic bifurcation. An important consideration when offering endovascular therapy in these cases is the normal aortic diameter, which can present gate cannulation challenges, especially in patients with a small aorta. To overcome this pitfall, we used a precannulation and snare-ride technique described by Mirza et al.^14^

Our patient has not had a new PE since being initiated on apixaban and was offered but refused filter removal. It is unclear whether, left in place, the filter can erode into graft material overtime. For this reason, continued surveillance after endovascular or open aortic reconstruction is imperative in cases where filters are not retrieved. Although the exact follow-up interval remains unknown, a noncontrast CT scan or ultrasound examination every other year or so seems reasonable.

No consensus exists on the management of patients with asymptomatic aortic wall penetration by IVCF struts; some, but not all, 93 patients with this complication reported in 31 identified publications underwent aortic surgery (Fig 1). The decision to repair varied greatly and seems to be left to the surgeon's discretion. However, anecdotal evidence strongly favors continued surveillance, at a minimum, because the majority of patients discussed in this report were followed for an asymptomatic aortic wall penetration by the IVCF strut that eventually progressed to APA. The time interval from filter placement to pseudoaneurysm development ranged from 9 months to 10 years (Table). Although lifelong anticoagulation has its own inherent complications, an IVCF should not be the first-line treatment for DVT/PE and should be removed as soon as possible to avoid long-term complications.^15^

## Conclusions

Endovascular repair of APA with a bifurcated stent graft is feasible and seems to be safe. However, whether performed open or endovascularly, continued surveillance is required especially when the filter is not retrieved as struts long-term impact on the graft material remains unknown. Further research on management of asymptomatic aortic wall penetration with IVCF struts is needed to guide clinical practice.
